# Prevalence of Heavy Menstrual Bleeding and Its Associated Cognitive Risks and Predictive Factors in Women With Severe Mental Disorders

**DOI:** 10.3389/fphar.2022.904908

**Published:** 2022-07-13

**Authors:** Jianmin Shan, Hongjun Tian, Chunhua Zhou, Haibo Wang, Xiaoyan Ma, Ranli Li, Haiping Yu, Guangdong Chen, Jingjing Zhu, Ziyao Cai, Chongguang Lin, Langlang Cheng, Yong Xu, Sha Liu, Congpei Zhang, Qinghua Luo, Yunshu Zhang, Shili Jin, Chuanxin Liu, Qiuyu Zhang, Luxian Lv, Lei Yang, Jiayue Chen, Qianchen Li, Wei Liu, Weihua Yue, Xueqin Song, Chuanjun Zhuo, China; MODMD Group of China (CMODG)

**Affiliations:** ^1^ Department of Psychiatry, Tianjin Fourth Center Hospital, Tianjin, China; ^2^ Department of Psychiatry, Wenzhou Seventh Peoples Hospital, Wenzhou, China; ^3^ Department of Pharmacology, The First Hospital of Hebei Medical University, Shijiazhuang, China; ^4^ Peking University Clinical Research Institute, Peking University First Hospital, Beijing, China; ^5^ MECT Center, Sleep Disorder Center, Tianjin Anding Hospital, Tianjin, China; ^6^ Department of Psychiatry, First Hospital/First Clinical Medical College of Shanxi Medical University, Taiyuan, China; ^7^ Inpatient Department of Harbin First Psychiatry Hospital, Harbin, China; ^8^ Department of Psychiatry, The First Affiliated Hospital of Chongqing Medical University, Chongqing, China; ^9^ Inpatient Department of Hebei Mental Health Center, Baoding, China; ^10^ Inpatient Department, Shandong Daizhuang Hospital, Jining, China; ^11^ Institute of Psychiatry, Jining Medical University, Jinning, China; ^12^ Department of Psychiatry, Henan Psychiatry Hospital, Xinxiang, China; ^13^ Key Laboratory of Mental Health, Ministry of Health (Peking University) and National Clinical Research Center for Mental Disorders (Peking University Sixth Hospital), Beijing, China; ^14^ Department of Psychiatry, The First Hospital Affiliated to Harbin Medical University, Harbin, China; ^15^ Department of Psychiatry, The First Affiliated Hospital of Zhengzhou University, Zhengzhou, China; ^16^ Laboratory of Psychiatric-Neuroimaging-Genetic and Cor-morbidity, Tianjin Mental Health Center of Tianjin Medical University, Tianjin Anding Hospital, Tianjin, China

**Keywords:** bipolar disorder, major depressive disorder, schizophrenia, heavy menstrual bleeding, visual learning, lithium

## Abstract

There has been limited studies examining treatment-induced heavy menstrual bleeding (HMB) in women with severe mental illnesses. The aim of this study was to examine HMB prevalence and HMB-associated factors in young women (18–34 years old) diagnosed with bipolar disorder (BP), major depressive disorder (MDD), or schizophrenia (SCZ) who have full insight and normal intelligence. Eighteen-month menstruation histories were recorded with pictorial blood loss assessment chart assessments of HMB. Multivariate analyses were conducted to obtain odds ratios (ORs) and 95% confidence intervals (CIs). Drug effects on cognition were assessed with the MATRICS Consensus Cognitive Battery (MCCB). HMB prevalence were: BP, 25.85%; MDD, 18.78%; and SCH, 13.7%. High glycosylated hemoglobin (HbA1c) level was a strong risk factor for HMB [BP OR, 19.39 (16.60–23.01); MDD OR, 2.69 (4.59–13.78); and SCZ OR, 9.59 (6.14–12.43)]. Additional risk factors included fasting blood sugar, 2-h postprandial blood glucose, and use of the medication valproate [BP: OR, 16.00 (95%CI 12.74–20.22); MDD: OR, 13.88 (95%CI 11.24–17.03); and SCZ OR, 11.35 (95%CI 8.84–19.20)]. Antipsychotic, antidepressant, and electroconvulsive therapy use were minor risk factors. Pharmacotherapy-induced visual learning impairment was associated with HMB [BP: OR, 9.01 (95%CI 3.15–13.44); MDD: OR, 5.99 (95%CI 3.11–9.00); and SCZ: OR, 7.09 (95%CI 2.99–9.20)]. Lithium emerged as a protective factor against HMB [BP: OR, 0.22 (95%CI 0.14–0.40); MDD: OR, 0.30 (95%CI 0.20–0.62); and SCZ: OR, 0.65 (95%CI 0.33–0.90)]. In SCZ patients, hyperlipidemia and high total cholesterol were HMB-associated factors (ORs, 1.87–2.22). Psychiatrist awareness of HMB risk is concerningly low (12/257, 2.28%). In conclusion, prescription of VPA should be cautioned for women with mental illness, especially BP, and lithium may be protective against HMB.

## Introduction

There is a relatively high prevalence of serious mental disorders, namely bipolar disorder (BP; lifetime prevalence, 2.4%), major depressive disorder (MDD; lifetime prevalence, 9.9%), and schizophrenia (SCZ, lifetime prevalence, 1%) in young women 18–34 years old (Kennedy et al., 2014; Tondo et al., 2014; Hui Poon et al., 2015; APA, 2016; Barnett, 2018; McIntyre et al., 2020; Howes et al., 2021; Papp et al., 2021; Rybak et al., 2021). Currently, they are treated primarily with antipsychotic agents, mood stabilizers (not a true pharmacological category; Stahl, 2021), antidepressants (Sienaert et al., 2013; Sinclair et al., 2019; Baandrup, 2020; Elias et al., 2021; Seppälä et al., 2021; Borbély et al., 2022), and electroconvulsive therapy (ECT) (Sinclair et al., 2019; Elias et al., 2021; Trifu et al., 2021).

Most antipsychotics inhibit serotonin reuptake, which can alter functioning of the hypothalamic-pituitary-gonadal (HPG) axis and thus may alter prolactin secretion and thereby cause menstrual disturbances (Huhn et al., 2019; Solmi et al., 2020), including heavy menstrual bleeding (HMB), hypomenorrhea, or amenorrhea (Haddad and Wieck, 2004; Kumar et al., 2013; Besag et al., 2021). Furthermore, HPG axis dysfunction can also cause glucose and lipid metabolism disorders that can lead to excessive weight gain and obesity (An et al., 2018; Tian et al., 2021; Piatoikina et al., 2022). Obesity is associated with dysregulation of adipose tissue functions and aberrations in adipokine secretion that can alter inflammatory responses, endothelial cell functions, and coagulation pathways.

Antipsychotic agents, antidepressant agents, and mood stabilizers have been reported to increase risk of hemorrhage and bleeding (Aranth and Lindberg, 1992; Joffe et al., 2006a; Paavola et al., 2019; Thomas et al., 2020). In women with severe mental illness, hemorrhage/bleeding presents mainly as HMB (El-Nashar et al., 2010; Stahl, 2021). HMB, which is defined as excessive regular or irregular menstrual bleeding (>80 ml per cycle) (Reavey et al., 2021b), affects 4% of women without organic pathology (Lethaby et al., 2009; Davies and Kadir, 2017). In mentally ill women, HMB has been reported to be associated with further psychiatric deterioration (Iles and Gath, 1989; Shannon, 1993; Pramodh, 2020; Arias-de la Torre et al., 2021; Padda et al., 2021). HMB in overweight women specifically has been related to metabolic disorders of blood glucose, lipids, and reproductive hormones (Iles and Gath, 1989; Shannon, 1993; Noerpramana, 1997; Pottegård et al., 2018; Pramodh, 2020; Schlaff et al., 2020; Arias-de la Torre et al., 2021; Padda et al., 2021; Pavlidi et al., 2021), all of which can be induced or exacerbated by psychiatric drugs (Iles and Gath, 1989; APA, 1994; Noerpramana, 1997; Meyer, 2002; Reynolds et al., 2007; Muir et al., 2008; Bradley and Gueye, 2016; Bora et al., 2017; Culpepper et al., 2017; Solé et al., 2017; Bekhbat et al., 2018; Pottegård et al., 2018; Calaf et al., 2020; Pramodh, 2020; Schlaff et al., 2020; Jang et al., 2021; Padda et al., 2021; Pavlidi et al., 2021).

Moreover, HMB can impair cognitive ability (Iles and Gath, 1989; Shannon, 1993; APA, 1994; Noerpramana, 1997; Meyer, 2002; Reynolds et al., 2007; Muir et al., 2008; El-Nashar et al., 2010; Bradley and Gueye, 2016; Bora et al., 2017; Culpepper et al., 2017; Solé et al., 2017; Bekhbat et al., 2018; Pottegård et al., 2018; Calaf et al., 2020; Pramodh, 2020; Schlaff et al., 2020; Arias-de la Torre et al., 2021; Jang et al., 2021; Padda et al., 2021; Pavlidi et al., 2021; Stahl, 2021), which may also be directly affected by mental illness and mental illness treatments (Iles and Gath, 1989; Shannon, 1993; APA, 1994; First et al., 1996; Noerpramana, 1997; Meyer, 2002; Reynolds et al., 2007; Muir et al., 2008; El-Nashar et al., 2010; Bradley and Gueye, 2016; Bora et al., 2017; Culpepper et al., 2017; Solé et al., 2017; Bekhbat et al., 2018; Pottegård et al., 2018; Calaf et al., 2020; Pramodh, 2020; Schlaff et al., 2020; Arias-de la Torre et al., 2021; Jang et al., 2021; Padda et al., 2021; Pavlidi et al., 2021; Stahl, 2021). Additionally, HMB may compromise patients’ reproductive potential (Iles and Gath, 1989; Shannon, 1993; APA, 1994; First et al., 1996; Noerpramana, 1997; Meyer, 2002; Reynolds et al., 2007; Muir et al., 2008; Bradley and Gueye, 2016; Bora et al., 2017; Culpepper et al., 2017; Solé et al., 2017; Bekhbat et al., 2018; Pottegård et al., 2018; Calaf et al., 2020; Pramodh, 2020; Schlaff et al., 2020; Arias-de la Torre et al., 2021; Jang et al., 2021; Padda et al., 2021; Pavlidi et al., 2021; Stahl, 2021). Hence, there is a multitude of negatively interacting factors related to HMB that can worsen the prognosis of young women suffering from serious mental disorders.

In the present study, we examined HMB occurrence and HMB-associated factors in young women diagnosed with BP, MDD, or SCZ who were being treated with antipsychotic medications. We conducted a retrospective multi-hospital study across 10 provinces in China. Data acquired for an 18-months target period were subjected to multiple regression analysis to investigate HMB-associated risk and protective factors in patients using therapeutic agents. The main aims of this work were to determine HMB prevalence, to identify HMB-associated associated factors in young women with severe mental illness, and to assess how aware psychiatrists are of HMB risk in this patient population.

## Materials and Methods

### Study Design and Participants

In this retrospective cohort study, we used convenience sampling to recruit participants being treated by 514 psychiatrists (257 senior psychiatrists, i.e., >10 years’ experience with annual research methods training) in outpatient departments at 10 hospitals located in the north, south, east, and west regions of China (across 10 provinces). A group of 10 gynecologists was invited to help assess HMB. The physician recruitment period lasted 2 months (July 1st to 31 August 2021). Recruited doctors furnished detailed information of the samples, including sociodemographic characteristics, diagnosis, menstrual cycle timing history from 1 January 2020 through 31 August 2021, HMB incidence, and cumulative therapeutic agent dosages. Informed consent forms were signed by patients and their guardians prior to data collection. Ethics approval was granted from the ethic committee of Tianjin Fourth Center Hospital of Tianjin Medical University (No. ZC-R-0001).

The patient inclusion criteria were as follows: 1) 18–34-year-old female patient with treatment-resistant BP, treatment-resistant MDD, or treatment-resistant SCZ; 2) first episode; 3) full insight about one’s own mental illness and treatment methods; 4) normal memory ability (to ensure recall of periods in the past 18 months); 5) medical record available to assure the absence of neurological or physical disease comorbidity, any history of menstrual dysfunction, and pharmacotherapies administrated in the prior 18 months; 6) willingness to volunteer participation in this study and provide detailed personal sociodemographic information. The exclusion criteria were as follows: 1) did not volunteer to participate; 2) cannot recall menstrual history of the past 18 months; 3) history of pregnancy and/or abortion in the past 18 months; 4) neurological illness, physical disease, or substance abuse history in the past 18 months; 5) diagnosis with any other mental disorder (including comorbid anxiety, depression, or personality disorder); 6) no majorly stressful life events in the past 18 months; and 7) no female family member/guardian available to assist with collecting information about the patient’s illness, menstrual status, HMB status, and other needed information. Typically, in Chinese culture, even if a woman has a close relationship with her husband, her mother will continue to manage her care from childhood into adulthood, which worked well for information acquisition in this study.

### Procedures

#### Data Collection

We collected clinical information from one insurance settlement period in China (3 months). Each participating physician collated the following patient information: category of mental illness; total menstrual cycles in the past 18 months; HMB incidence in the past 18 months; total cumulative medication dosage in the past 18 months; glycosylated hemoglobin A1c (HbA1c) level; steroid hormone levels; blood sugar levels; blood total cholesterol (TC) levels; blood lipid levels; and body mass index (BMI). BP, MDD, and SCZ definitions were adopted from the Diagnostic and Statistical Manual of Mental Disorders–Edition IV (First et al., 1996) and Structured Clinical Interview for DSM-IV Axis I Disorders (Birchwood et al., 1994). Mental illness etiology was described based on core symptoms. Blood constituent level data determined closest to the start of the study were used. Each patient’s medical record was consulted for confirming medication dosage accuracy and an absence of neurological and physical disease history in the past 18 months.

#### Instruments

Patient insight was confirmed with the Birchwood Insight Scale (Beck et al., 2004) and Beck Cognitive Insight Scale (Wang et al., 2015). Normal memory function was confirmed with the Chinese version of the Wechsler memory scale-Fourth edition (Ko et al., 2021).

Total numbers of menstrual times in the past 18 months were recorded and the Pictorial Blood-loss Assessment Chart (PBAC) (Leucht et al., 2003) was used to assess HMB (although PBAC is used extensively to assess HMB at the moment, its validity regarding previous HMB status remains to be verified). Because this study was conducted retrospectively, we brought in gynecologists to show patients and their female guardians HMB on PBAC and guide information collection, including the presence of blood clots on sanitary napkins, how many sanitary napkins were used in one menstrual period, and how to calculate menstrual bleeding. Although previous studies have used more accurate HMB diagnostic criteria, the large sample included in this study precluded the use of such a highly complex procedure. Although our retrospective use of the PBAC represents a limitation, it provided useful information for this study. Eighteen-month cumulative antipsychotic, antidepressant, mood stabilizer, and anxiolytic/sleep aide use were converted to chlorpromazine equivalent (Hayasaka et al., 2015), fluoxetine equivalent (Rossetti and Alvarez, 2021), sodium valproate equivalent (García-Carmona et al., 2021), and diazepam equivalent (Paulzen et al., 2016), respectively.

Obesity was determined based on BMI (Mizuno et al., 2014). Prolactin, estradiol, progesterone, testosterone levels were determined with double-antibody radio-immunoassays. The cut-offs for hyperprolactinemia, high progesterone, and high testosterone were ≥40.0 μg/L (Nikolac Gabaj et al., 2018), ≥97.6 nmol/L, and ≥3.1 nmol/L (Lee et al., 2013), respectively. Fasting and 2-h postprandial blood glucose analyses (Gu et al., 2016) were conducted by a Cobas 6,000 analyzer (Roche Diagnostics GmbH, Mannheim, Germany). Glycosylated hemoglobin (HbA1c), TC, and blood lipid levels (Nick and Campbell, 2007) were determined with an H-7600 automated biochemical analyzer (Hitachi High-Technologies, Tokyo, Japan).

Pregnancy tests were conducted to exclude pregnant patients from the study (Wang et al., 2015). An HMB self-report scale was used to assess patients’ views of the influence of HMB on their mental illness status. It included the following items and answer point values: 1) did you feel your mental illness symptoms deteriorated from HMB? (yes, 1; no, 0); 2) did the total deteriorated time exceed a month? (yes, 1; no, 0 AND if severe, +3, if moderate +2, if mild +1); 3) did you feel that you were suffering when experiencing HMB? (yes, 1; no, 0); 4) did you feel the total time of your suffering from HMB exceeded a month? (yes, 1; no, 0; severe suffering, +3; moderate suffering, +2; mild suffering, +1); 5) did you feel your life quality in the past 18 months was influenced by your HMB? (severe influence, four; moderate influence, three; mild influence, two; no influence, 0).

Cognition was assessed with the MATRICS Consensus Cognitive Battery (MCCB), which has a three-domain structure (McCleery et al., 2015; Lo et al., 2016). Specifically, MCCB assessments of processing speed (Trail Making Test, Part A; Symbol Coding; and Category Fluency: Animal Naming), working memory (Letter-Number Span); and verbal learning (Hopkins Verbal Learning Test-Revised; HVLT-R) were conducted. Verbal learning was assessed with the Rey Auditory Verbal Learning Test (Schmidt, 1996; Bowie et al., 2018) instead of the HVLT-R in one participant.

### Statistical Analysis

Statistical analyses were completed in SAS statistical software (version 9.3, SAS Institute, Cary, NC). Continuous-variable data are expressed as means ± standard deviations (normally distributed data). Data are compared across groups and within groups over time with analyses of variance (ANOVAs) and repeated measures ANOVAs, respectively. Categorical-variable data are expressed as numbers and percentages. Associations of clinical-demographic characteristics with HMB incidence were evaluated with univariate and multivariate logistic regression models and expressed as odds ratios (ORs) and 95% confidence intervals (CIs) in the overall sample and by diagnosis group. Multivariate logistic models were first developed by adjusting for factors found to be significant in univariate analyses (*p* < 0.02); final multivariate models were limited to risk factors or confounders that were statistically significant (Nikoloulopoulos, 2012; Hidalgo and Goodman, 2013; Vuong et al., 2014; Fu et al., 2020).

## Results

### Sample

Complete information was obtained 3,094 of 3,500 participants (88.4%) who were recruited to enroll in this study. The final study sample of 3,094 participants, included 855 patients with BP, 1,049 patients with MDD, and 1,190 patients with SCZ. The BP, MDD, and SCZ groups were similar with respect to age, education level, and illness duration. The sample characteristics (as a whole and of each diagnosis group) are summarized in Table 1.

**TABLE 1:** Characteristics of the total sample (*N* = 3,094) and the bipolar disorder (BP; *N* = 855), major depressive disorder (MDD; *N* = 2,049), and schizophrenia (SCZ; *N* = 1, 190) groups.

Variable		BP	MDD	SCZ	All
Education y, >12		557 (65.1)	556 (53.0)	664 (55.8)	1,777 (57.4)
Age, y		27.9 ± 3.7	27.6 ± 3.9	27.7 ± 3.8	27.7 ± 3.8
Total illness duration, mos		39.5 ± 9.0	41.1 ± 8.8	41.4 ± 9.0	40.8 ± 9.0
Pre-treatment BMI		20.8 ± 2.5	21.9 ± 2.2	22.5 ± 2.6	21.8 ± 2.5
First seeking treatment	HbA1c, %	4.2 ± 0.1	4.3 ± 0.2	4.1 ± 0.5	4.2 ± 0.6
	FBS, mmol/L	4.7 ± 0.4	4.9 ± 0.1	4.8 ± 0.2	4.8 ± 0.7
	PBG-2h, mmol/L	6.2 ± 1.1	6.9 ± 0.7	7.1 ± 1.5	6.9 ± 0.2
	TC, mmol/L	2.3 ± 0.3	2.0 ± 0.4	2.2 ± 0.4	2.2 ± 0.2
	TG, mmol/L	1.2 ± 0.1	1.4 ± 0.2	1.5 ± 0.3	1.3 ± 0.3
	Prolactin, ng/ml	8.1 ± 2.9	6.6 ± 2.3	9.9 ± 2.9	8.3 ± 3.0
	Estradiol, pg/ml	788.7 ± 370.2	695.9 ± 299.3	680 ± 303.9	700.5 ± 317.3
	Prog, ng/ml	13.8 ± 5.7	14.5 ± 6.0	13.8 ± 25.2	13.2 ± 4.6
	Testos, nmol/L	0.8 ± 0.3	1.0 ± 0.4	1.9 ± 0.9	1.5 ± 0.7
1–2 mos after accepting treatment	HbA1c, %	4.6 ± 0.5	4.4 ± 0.6	4.7 ± 0.5	4.6 ± 0.5
	FBS, mmol/L	5.5 ± 0.2	5.3 ± 0.4	5.7 ± 0.3	5.5 ± 0.5
	PBG-2h, mmol/L	6.1 ± 1.0	6.0 ± 0.7	6.2 ± 0.9	6.1 ± 0.5
	TC, mmol/L	3.7 ± 0.5	3.2 ± 0.4	3.9 ± 0.8	3.6 ± 0.1
	TG, mmol/L	1.7 ± 0.3	1.4 ± 0.2	1.9 ± 0.5	1.8 ± 0.1
	Prolactin, ng/ml	513.6 ± 230.9	355.6 ± 145.5	694.8 ± 241	529.7 ± 255.1
	Estradiol, pg/ml	696.5 ± 88.7	438.7 ± 110.2	490.6 ± 138.8	390.5 ± 117.3
	Prog, ng/ml	18.9 ± 2.2	25.4 ± 4.9	22.7 ± 6.5	21.8 ± 3.9
	Testos, nmol/L	1.5 ± 0.4	1.9 ± 0.7	2.3 ± 1.1	2.0 ± 0.5
2–3 mos after accepting treatment	HbA1c, %	5.6 ± 0.4	5.1 ± 0.3	5.6 ± 0.3	5.2 ± 0.5
	FBS, mmol/L	6.0 ± 0.2	5.6 ± 0.3	6.1 ± 0.4	5.9 ± 0.5
	PBG-2h, mmol/L	9.2 ± 0.2	8.5 ± 0.1	9.5 ± 0.4	9.4 ± 0.5
	TC, mmol/L	4.2 ± 0.4	3.9 ± 0.5	4.1 ± 0.9	4.0 ± 0.1
	TG, mmol/L	2.8 ± 0.2	2.1 ± 0.1	2.9 ± 0.5	2.6 ± 0.3
	Prolactin, ng/ml	1,528.0 ± 289.8	1,230.9 ± 251.6	1,883.0 ± 293.7	1,607.4 ± 318.6
	Estradiol, pg/ml	662.3 ± 202.4	398.3 ± 118.9	369.7 ± 220.0	525.40 ± 125.5
	Prog, ng/ml	26.0 ± 9.7	28.6 ± 6.9	30.4 ± 5.2	29.5 ± 10.2
	Testos, nmol/LL	2.2 ± 0.2	2.3 ± 0.3	2.7 ± 0.5	2.6 ± 0.8
Study enrollment	HbA1c, %	5.7 ± 0.3	5.4 ± 0.1	5.8 ± 0.2	5.5 ± 0.4
	FBS, mmol/L	6.3 ± 0.2	6.0 ± 0.1	6.4 ± 0.2	6.3 ± 0.3
	PBG-2h, mmol/L	9.1 ± 0.5	8.8 ± 0.5	9.3 ± 0.6	9.1 ± 0.9
	TC, mmol/L	5.3 ± 0.6	5.2 ± 0.3	5.8 ± 0.2	5.5 ± 0.5
	TG, mmol/L	2.9 ± 0.4	2.3 ± 0.5	3.1 ± 0.5	2.9 ± 0.8
	Prolactin, ng/ml	2,158.6 ± 797.2	1754.4 ± 627.7	2,628.3 ± 1101.7	2,200.5 ± 957.1
	Estradiol, pg/ml	541.8 ± 155.7	349.8 ± 158.6	362.8 ± 244.4	444.71 ± 97.85
	Prog, ng/ml	29.5 ± 12.3	33.2 ± 15	36.6 ± 20.4	28.9 ± 11.5
	Testos, nmol/L	2.9 ± 1.0	3.5 ± 0.9	3.4 ± 1.5	3.0 ± 1.7
MD ≤ 1 year pre-illness onset	No	441 (51.6)	901 (85.9)	1052 (88.4)	2,394 (77.4)
	Yes	414 (48.4)	148 (14.1)	138 (11.6)	700 (22.6)
HMB ≤1 year pre-illness onset	No	756 (88.4)	1,001 (95.4)	1145 (96.2)	2,902 (93.8)
	Yes	99 (11.6)	48 (4.6)	45 (3.8)	192 (6.2)
No. HMB periods in the year		1.8 ± 1.3	2.0 ± 1.3	1.6 ± 1.3	1.8 ± 1.3
*Pre-treatment MCCB*		BP	MDD	SCZ	ANOVA *p*
Speed procession		35.30 ± 5.45	35.14 ± 4.25	34.07 ± 2.30	0.667
Attention vigilance		36.47 ± 6.25	36.55 ± 3.12	37.53 ± 3.02	0.577
Working memory		36.65 ± 4.30	36.57 ± 2.39	38.22 ± 3.20	0.690
Verbal learning		37.20 ± 2.77	37.68 ± 2.24	38.45 ± 2.87	0.735
Visual learning		36.56 ± 6.23	36.09 ± 3.12	37.03 ± 1.88	0.702
Reasoning		37.25 ± 2.58	39.24 ± 3.38	39.97 ± 3.69	0.825
Social recognition		38.99 ± 5.84	37.15 ± 3.69	32.93 ± 1.78	0.920
Composite		30.03 ± 2.70	31.26 ± 3.09	30.99 ± 3.76	0.911

Mos, months; BMI, body mass index; HbA1c, hemoglobin A1C; FBS, fasting blood glucose; PBG-2h, 2-h postprandial blood glucose; TC, total cholesterol; TG, triglycerides; Prog, progesterone; Testos, testosterone; MD, menstrual dysfunction; HMB, heavy menstrual bleeding; MCCB, MATRICS, consensus cognitive battery; ANOVA, analysis of variance.

The patients’ treatment histories, including their interactions with their physicians in relation to HMB awareness as indicated on the HMB self-report scale, are summarized in Table 2, respectively. Although the patients indicated that HMB had negative effects on their illness progression and quality of life, few patients had been informed that HMB was a potential secondary adverse reaction to psychiatric medications. Furthermore, when queried, only 12 of the 257 psychiatrists servicing these patients (awareness rate, 2.28%) were aware of the risk.

**TABLE 2:** Treatment information within the 18 months before enrolling in the study.

Variable		BP	MDD	SCZ	All
Chlorpromazine equivalent, mg		116,983.4 ± 61,504.3	124,359.1 ± 68,115.2	324,041.0 ± 94,638.3	275,796.7 ± 123,914.7
Fluoxetine equivalent, mg		2,046.78 ± 6,813.3	21,161.7 ± 3,540.89	18,560.8 ± 6,006.9	20,328.2 ± 534.0
Valproate equivalent, mg		973,003.5 ± 613,025	274,344.0 ± 210,271.5	448,888.5 ± 280,239.7	563,127.9 ± 232,708.7
Total lithium, mg		406,189.2 ± 4,808.5	364,447.4 ± 2,756.3	102,222.5 ± 1,200.8	275,402.3 ± 2,735.4
Diazepam equivalent, mg		8,836.8 ± 2,648.2	8,627.7 ± 2,691.4	8,240.8 ± 2,727.9	8,539.5 ± 2,701.9
Benzhexol total dosage, mg		2,776.1 ± 544.7	2,861.3 ± 539	2,729.7 ± 547.4	2,763.8 ± 544.6
Promethazine total dosage, mg		49,050.0 ± 28,059.5	24,080.4 ± 12,350.7	55,233.0 ± 33,039.9	53,473.9 ± 31,767.9
Cumulative aripiprazole dose (hyperprolactinemia treatment)		2,907.4 ± 681.4	2,918.4 ± 700.2	2,996.6 ± 816.6	2,946.3 ± 744.5
*HMB self-report scale responses, mean ± standard deviation or N (%)*					
No. menstrual cycles		6.6 ± 2.3	5.3 ± 2.6	6.5 ± 1.9	6.1 ± 2.3
HMB	No	634 (74.15)	852 (81.22)	1,027 (86.3)	2,513 (71.22)
	Yes	221 (25.85)	197 (18.78)	163 (13.70)	581 (18.78)
HMB frequency	4.1 ± 1.1	2.4 ± 0.5	1.5 ± 0.3	2.2 ± 0.6	
Accepted ECT	No	530 (62.0)	684 (65.2)	592 (49.7)	1,806 (58.4)
	Yes	325 (38.0)	365 (34.8)	598 (50.3)	1,288 (41.6)
No. ECT sessions		36.0 ± 11.8	35.0 ± 11.9	35.5 ± 12.5	35.5 ± 12.1
HMB-related anemia treatment	No	137 (61.99)	154 (78.17)	135 (82.83)	426 (67.15)
	Yes	84 (38.01)	43 (21.83)	28 (17.17)	155 (32.85)
Nutrition change only for HMB-related anemia	No	0 (0.0)	0 (0.0)	0 (0.0)	0 (0.0)
	yes	137 (100)	154 (100)	135 (100)	426 (100)
Patient knows HMB is a side effect of drugs	No	842 (98.5)	1,035 (98.7)	1,176 (98.8)	3,053 (98.7)
	Yes	13 (1.5)	14 (1.3)	14 (1.2)	41 (1.3)
Doctor said HMB is a side effect of drugs	No	853 (99.76)	1046 (98.7)	1,183 (98.8)	3,041 (98.7)
	Yes	2 (0.24)	3 (0.29)	7 (0.60)	12 (0.39)
Pharmacotherapy for menstrual dysfunction	No	810 (94.74)	977 (93.14)	245 (20.59)	2,032 (98.7)
	Yes	45 (5.26)	72 (6.86)	945 (79.41)	1062 (2.3)
TCM for menstrual dysfunction	No	199 (23.27)	260 (24.79)	340 (29.40)	799 (26.72)
	Yes	656 (76.73)	789 (75.21)	850 (71.60)	2,295 (74.18)
Doctor asks about HMB and adjusts treatment to alleviate it	No	853 (99.76)	1,046 (98.7)	1,183 (98.8)	3,041 (98.7)
	Yes	2 (0.24)	3 (0.29)	7 (0.60)	12 (0.39)
Illness deteriorated due to HMB; if yes, degree rating	No	0 (0)	0 (0)	0 (0)	0 (0)
	<15%	25 (11.31)	33 (16.75)	35 (21.47)	93 (14.98)
	15%–30%	37 (16.74)	29 (14.72)	37 (22.70)	103 (16.59)
	30%–50%	41 (18.55)	37 (18.78)	25 (15.15)	103 (16.59)
	≥50%	118 (53.39)	98 (49.74)	66 (40.49)	282 (45.41)
Told doctor about HMB-related anemia treatment	No	204 (92.31)	175 (88.32)	155 (95.09)	534 (91.91)
	Yes	17 (7.69)	22 (11.68)	8 (4.91)	47 (8.09)
After telling, doctor adjusted meds or advised protection	No	204 (92.31)	175 (88.32)	155 (95.09)	534 (91.91)
	Yes	17 (7.69)	22 (11.68)	8 (4.91)	47 (8.09)

Questionnaire responses are presented as N(%). HMB, heavy menstrual bleeding; ECT, electroconvulsive therapy; TCM, traditional chinese medicine.

### HMB Prevalence

It was determined that 581/3,094 participants (18.78%) had HMB. Their average frequency of HMB in the past 18 months was 2.2 ± 0.6 menstrual cycles. The HMB prevalence rates by diagnosis group were: BP, 28.85% (221/855); MDD, 18.78% (197/1.49); and SCZ, 13.70% (163/1190). The prevalence rates of HMB in patients with BP and patients with MDD were 2.52-fold and 1.21-fold that in patients with SCZ.

### Evolution of Cognitive Functioning

Mean (±standard deviations) of MCCB scores over time are reported in Table 3. Note that no MCCB domain scores differed between the diagnosis groups prior to the patients starting pharmacological treatment, though differences emerged over time, with diagnosis group having a main effect on all MCCB domain scores, except working memory, by the time of the study enrollment assessment. Visual learning was the most heavily impacted domain (see Table 3). Repeated measures ANOVAs showed that all MCCB domain scores changes over time for all groups, though the composite scores changed significantly over time for only the BP group.

**TABLE 3:** Comparison of MCCB scores before treatment, after 3 months treatment, and at study enrollment.

*Time of assessment* MCCB dimension	Diagnosis group			Inter-group ANOVA *p*
	BP	MDD	SCZ	
*Before treatment*				
Speed procession	35.30 ± 5.45	35.14 ± 4.25	34.07 ± 2.30	0.667
Attention vigilance	36.47 ± 6.25	36.55 ± 3.12	37.53 ± 3.02	0.577
Working memory	36.65 ± 4.30	36.57 ± 2.39	38.22 ± 3.20	0.690
Verbal learning	37.20 ± 2.77	37.68 ± 2.24	38.45 ± 2.87	0.735
Visual learning	36.56 ± 6.23	36.09 ± 3.12	37.03 ± 1.88	0.702
Reasoning	37.25 ± 2.58	39.24 ± 3.38	39.97 ± 3.69	0.825
Social recognition	38.99 ± 5.84	37.15 ± 3.69	32.93 ± 1.78	0.920
Composite	30.03 ± 2.70	31.26 ± 3.09	30.99 ± 3.76	0.911
*2 ∼ 3 months from accepting 1*st *treatment*				
Speed procession	30.28 ± 3.69	29.87 ± 7.35	26.02 ± 1.82	0.051
Attention vigilance	30.66 ± 2.56	31.99 ± 1.75	30.36 ± 1.82	0.361
Working memory	30.33 ± 4.75	32.11 ± 1.87	30.25 ± 1.52	0.123
Verbal learning	32.55 ± 2.55	30.99 ± 1.98	32.00 ± 1.25	0.317
Visual learning	20.21 ± 1.22	20.00 ± 1.45	20.03 ± 1.07	0.675
Reasoning	32.15 ± 2.99	34.05 ± 1.39	30.04 ± 1.85	0.049
Social recognition	31.22 ± 1.79	28.88. ± 3.23	25.44 ± 3.45	0.037
Composite	29.44 ± 1.52	30.21 ± 1.44	28.52 ± 2.13	0.063
*Study enrollment*				
Speed procession	24.77 ± 1.13	26.47 ± 1.05	24.78 ± 10.86	0.049
Attention vigilance	28.35 ± 0.85	28.23. ± 0.45	27.66 ± 1.10	0.024
Working memory	30.02 ± 0.45	30.51 ± 0.69	29.17 ± 1.23	0.335
Verbal learning	29.56 ± 1.17	28.96 ± 0.59	27.59 ± 1.37	0.046
Visual learning	16.44 ± 0.85	15.28 ± 0.78	13.25 ± 0.85	0.017
Reasoning	30.00 ± 0.77	31.25 ± 0.80	25.14 ± 1.11	0.022
Social recognition	30.57 ± 1.74	32.55 ± 0.98	28.00 ± 0.97	0.047
Composite	24.88 ± 1.25	28.00. ± 1.52	20.44 ± 0.35	0.010
*Intra-group rmANOVA*				
Speed procession	0.0297	0.0219	0.0401	—
Attention vigilance	0.0378	0.0347	0.0234	—
Working memory	0.0481	0.0301	0.0377	—
Verbal learning	0.0394	0.0285	0.0390	—
Visual learning	<0.0001	<0.0001	0.0001	—
Reasoning	0.0493	0.0313	0.0010	—
Social recognition	0.0019	0.0477	0.0473	—
Composite	0.0010	0.0599	0.211	—
*Score reduction*				
Speed procession	34.60%	34.69%	27.27%	—
Attention vigilance	22.26%	22.76%	36.79%	—
Working memory	18.59%	16.57%	37.46%	—
Verbal learning	20.73%	23.14%	28.44%	—
Visual learning	56.42%	57.66%	64.22%	—
Reasoning	17.58%	20.34%	39.34	—
Social recognition	37.5%	13.35%	14.97%	—
Composite	39.20%	10.43%	33.72%	—

MCCB, MATRICS, consensus cognitive battery; BP, bipolar disorder; MDD, major depressive disorder; SCZ, schizophrenia; ANOVA, analysis of variance; rmANOVA, repeated measures ANOVA.

### HMB-Associated Factors

As reported in detail in Table 4, univariate analyses indicated that the following variables were associated with HMB (ORs, 1.86–16.77): <30 years old; HbA1c; fasting blood sugar (FBS); and 2-h postprandial blood glucose (PBG-2h). As reported in detail in Table 5, multivariate analysis demonstrated that HMB in BP patients was associated with being younger than 30 years old as well as with visual learning scores, HbA1c levels, FBS, and PBG-2h within 2–3 months after commencing pharmacological treatment. In patients with SCZ, hyperlipidemia and high TC were associated with HMB. Reproductive hormone concentrations did not fluctuate in association with HMB. Notably, HMB was found to be strongly significantly associated with cumulative antidepressant, valproate, and lithium use in the patient sample as a whole and in each diagnosis group (Table 5), with the former two being a risk association (use predicts higher HMB risk) and the latter one being a protective association (use predicts lower HMB risk).

**TABLE 4:** Univariate analysis results.

Factor	Or (95%CI)				
		BP	MDD	SCZ	All
Diagnosis	SCZ	—	—	—	1.0
	MDD	—	—	—	1.04 (0.83–1.30)
	BP	—	—	—	1.66 (1.31–2.09)
Education	≤12 years	1.0	1.0	1.0	1.0
	>12 years	1.00 (0.71–1.41)	0.74 (0.54–1.00)	1.00 (0.72–1.39)	0.88 (0.73–1.06)
Age <30 years		5.93 (3.89–9.44)	3.45 (2.17–7.88)	1.96 (1.15–4.20)	3.33 (1.10–4.07)
Illness duration, mos		1.01 (0.99–1.03)	1.01 (0.99–1.03)	1.00 (0.98–1.02)	1.01 (1.00–1.02)
Pretreatment BMI		1.80 (0.95–4.41)	1.33 (0.78–3.39)	2.74 (2.33–3.21)	1.06 (0.36–1.10)
Before accepting 1st treatment		5.93 (3.89–9.44)	3.45 (2.17–7.88)	1.96 (1.15–4.20)	3.33 (1.10–4.07)
HbA1c, %		6.82 (4.80–9.68)	5.38 (3.85–7.51)	4.14 (2.94–5.84)	4.10 (3.43–4.91)
FBS, mmol/L		16.77 (10.81–26.02)	9.91 (6.67–14.71)	7.68 (4.93–11.95)	8.75 (6.94–11.02)
PBG-2h, mmol/L		4.71 (3.70–6.01)	1.86 (1.47–2.08)	2.66 (2.27–3.12)	2.50 (2.30–2.72)
TC, mmol/L		1.05 (0.83–1.33)	1.28 (1.03–1.59)	0.95 (0.75–1.21)	1.10 (0.96–1.25)
TG, mmol/L		0.76 (0.48–1.20)	0.69 (0.45–1.04)	0.75 (0.47–1.19)	0.74 (0.57–0.95)
Prolactin, ng/ml		0.99 (0.93–1.05)	1.02 (0.95–1.08)	0.98 (0.93–1.04)	1.02 (0.99–1.06)
Within 1–2 mos of accepting 1st treatment		6.82 (4.80–9.68)	5.38 (3.85–7.51)	4.14 (2.94–5.84)	4.10 (3.43–4.91)
HbA1c, %		29.66 (18.48–47.62)	5.83 (4.26–7.99)	6.17 (4.19–9.09)	7.20 (5.88–8.82)
FBS, mmol/L		22.21 (13.86–35.59)	11.18 (7.48–16.70)	6.17 (4.17–9.13)	10.13 (8.02–12.80)
PBG-2h, mmol/L		4.13 (3.32–5.14)	1.85 (1.66–2.07)	2.52 (2.18–2.92)	2.43 (2.25–2.63)
TC, mmol/L		0.88 (0.69–1.12)	0.97 (0.78–1.20)	1.07 (1.00–2.36)	0.98 (0.86–1.12)
TG, mmol/L		0.98 (0.62–1.56)	1.07 (0.71–1.62)	1.59 (1.64–7.57)	1.02 (0.79–1.32)
Prolactin, ng/ml		1.78 (0.95–4.20)	1.79 (0.98–6.82)	6.48 (2.08–10.70)	2.30 (0.85–5.35)
Within 2–3 months of accepting 1st treatment		29.66 (18.48–47.62)	5.83 (4.26–7.99)	6.17 (4.19–9.09)	7.20 (5.88–8.82)
HbA1c, %		5.17 (3.01–9.57)	8.96 (6.13–13.10)	7.90 (4.00–10.13)	5.92 (2.35–10.53)
FBS, mmol/L		5.42 (4.22–9.9)	9.08 (5.98–14.55)	4.59 (2.47–9.88)	3.59 (1.88–7.00)
PBG-2h, mmol/L		9.59 (6.91–13.30)	2.08 (1.82–2.38)	6.82 (5.22–8.92)	3.80 (3.41–4.24)
TC, mmol/L		1.24 (0.97–1.57)	0.86 (0.69–1.07)	4.10 (1.09–7.15)	1.01 (0.89–1.15)
TG, mmol/L		0.98 (0.62–1.56)	0.89 (0.58–1.36)	3.87 (1.98–9.75)	0.97 (0.75–1.26)
MD in year before this study	Yes	1.0	1.0	1.0	1.0
	No	1.78 (0.56–4.08)	1.16 (1.11–2.23)	1.34 (0.76–2.36)	1.49 (0.88–5.60)
HMB in year before this study	Yes	1.0	1.0	1.0	1.0
	No	1.18 (0.16–2.54)	0.82 (0.74–6.33)	1.04 (0.29–1.36)	1.00 (0.88–1.60)
ECT treatment 18mPreEn	Yes	1.0	1.0	1.0	1.0
	No	3.33 (2.22–5.01)	2.08 (1.47–2.96)	5.03 (3.35–7.54)	3.35 (2.69–4.18)
Cumulative dosage 18mPreEnr		2.66 (1.12–6.33)	1.85 (1.26–2.81)	1.77 (1.45–3.31)	2.22 (1.96–3.56)
Antipsychotic^a^		2.37 (1.48–3.78)	2.69 (1.06–6.83)	3.22 (1.18–8.19)	1.96 (1.34–2.86)
Antidepressant^a^		2.71 (1.10–7.35)	4.13 (2.70–8.81)	3.21 (1.90–5.40)	1.95 (1.76–5.19)
Anti-mania agent^a^		0.20 (0.12–0.49)	0.29 (0.17–0.48)	0.49 (0.20–0.87)	0.60 (0.31–0.99)
Mood stabilizer^a^		0.93 (0.39–2.20)	0.93 (0.44–1.98)	0.25 (0.10–0.64)	0.60 (0.37–0.97)
Anxiolytic^a^		1.46 (0.19–11.31)	0.27 (0.17–5.00)	2.65 (0.13–52.45)	1.18 (0.25–5.67)
Spasmolytic^a^		1.45 (0.75–2.79)	0.85 (0.42–1.59)	1.23 (0.83–1.83)	1.29 (0.92–1.80)
Anti-nausea/pain^a^		2.52 (0.65–4.80)	1.60 (0.85–2.46)	1.97 (0.67–4.05)	2.28 (0.73–5.25)
Antipsychotic^a^		1.27 (0.56–4.99)	0.89 (0.75–1.37)	1.44 (0.93–3.66)	1.11 (0.49–1.75)
TCM for MD		9.37 (3.18–14.56)	6.24 (4.18–9.43)	8.56 (7.14–13.25)	7.80 (3.13–29.99)
Visual learning, 2∼3mTx		1.57 (1.01–3.45)	1.07 (1.00–3.11)	2.00 (1.00–6.02)	1.47 (1.00–6.59)
Composite, 2∼3mTx		2.66 (1.12–6.33)	1.85 (1.26–2.81)	1.77 (1.45–3.31)	2.22 (1.96–3.56)

a Logarithms used in analysis and reported as follows: antipsychotic, chlorpromazine equivalent (eq); antidepressant, fluoxetine eq; anti-mania agent, valproate eq; mood stabilizer, lithium eq; anxiolytic, diazepam eq; spasmolytic, trihexyphenidyl eq; anti-nausea/pain, promethazine eq; antipsychotic, aripiprazole eq. OR, odds ratio; CI, confidence interval; BP, bipolar disorder; MDD, major depressive disorder; SCZ, schizophrenia; HbA1c, hemoglobin A1C; FBS, fasting blood glucose; PBG-2h, 2-h postprandial blood glucose; TC, total cholesterol; TG, triglycerides; MD, menstrual dysfunction; HMB, heavy menstrual bleeding; ECT, electroconvulsive therapy; 18mPreEn, within the 18 months before enrolling to participate in the study; TCM, traditional Chinese medicine; 2∼3mTx, within first 2–3 months of treatment.

**TABLE 5:** Multivariate analysis of HMB risk factors in each diagnosis group and in the total sample.

Factor	BP		SCZ		MDD		All	
	Or (95% CI)	*p*	Or (95% CI)	*p*	Or (95% CI)	*p*	Or (95% CI)	*p*
Age <30 years	4.89 (2.21–8.59)	<0.0001	1.87 (1.10–3.56)	<0.0001	3.12 (2.19–7.874)	<0.0001	2.87 (1.09–3.97)	<0.0001
HbA1c^a^	19.39 (16.60–23.01)	0.0012	2.33 (1.76–3.09)	<0.0001	12.69 (4.59–13.78)	<0.0001	2.89 (1.55–5.37)	0.0008
FBS^a^	15.87 (11.85–119.81)	<0.0001	9.59 (6.14–12.43)	<0.0001	9.97 (7.52–13.49)	<0.0001	40.87 (16.50–65.23)	<0.0001
PBG-2h^a^	9.22 (6.48–12.35)	0.0001	8.53 (6.15–12.61)	<0.0001	8.44 (4.63–13.44)	<0.0001	1.75 (1.33–2.29)	<0.0001
Visual learning^a^	14.12 (11.15–15.99)	<0.0001	13.19 (7.80–15.08)	<0.0001	10.00 (10.03–14.08)	<0.0001	6.58 (2.17–10.81)	0.0004
ECT treatment	3.11 (1.19–5.00)	0.0298	9.03 (7.15–12.30)	<0.0001	5.55 (2.94–12.87)	<0.0001	1.92 (1.13–3.26)	0.0167
*Cumulative dose*								
Antipsychotic	5.51 (3.89–11.52)	<0.0001	2.22 (1.23–4.55)	<0.0001	1.87 (1.30–4.10)	<0.0001	3.52 (1.58–12.15)	<0.0001
Antidepressant	1.14 (1.02–2.88)	<0.0001	1.87 (1.27–6.00)	<0.0001	9.03 (7.23–12.48)	<0.0001	2.28 (1.22–11.00)	<0.0001
Valproate	16.00 (12.74–20.22)	<0.0001	4.12 (2.66–9.50)	<0.0001	4.88 (1.24–7.03)	<0.0001	4.59 (2.85–9.60)	<0.0001
Lithium	0.22 (0.14–0.40)	<0.0001	11.99 (6.74–14.44)	<0.0001	0.30 (0.20–0.62)	<0.0001	0.50 (0.09–0.99)	<0.0001
*Group comparisons* BP vs. SCH: 2.52 (1.50–3.13), *p* < 0.0001 MDD vs. SCH: 1.21 (1.12–2.01), *p* = 0.0002								

a Within 2–3 months of accepting the first treatment. BP, bipolar disorder; SCZ, schizophrenia; MDD, major depressive disorder; OR, odds ratio; CI, confidence interval.

## Discussion

This study was the first to our knowledge to examine HMB risk factors in patients with severe mental illness. We found that nearly one in five of the women in our study experienced HMB. Treatment-related HMB was associated with mental illness symptom deterioration in a majority of those patients, with the symptom deterioration being severe for more than half of those affected and 17.17%–38.01% of patients with HMB needing a professional gynecological medical intervention. The destructive influence of HMB in these patients was most prevalent in women diagnosed with BP. Our analyses revealed additional valuable information in multiple areas important for quality of care as elaborated below.

In the present study sample, HMB was more prevalent in patients with BP than in patients with MDD or SCZ. The reasons for this differentiation are not known and worthy of exploring in future studies. We found that high blood sugar levels were associated with HMB risk in patients with BP. Patients with BP had higher rates of menstrual dysfunction in our sample than in several previous studies (Rasgon et al., 2005a; Rasgon et al., 2005b; Konicki et al., 2021). Half of the BP patients with HMB in our sample were diagnosed with menstrual dysfunction before being diagnosed with BP. Meanwhile 38% developed menstrual dysfunction only after starting psychiatric pharmacotherapy, ∼80% of whom experienced menstrual flow increases, including HMB or prolonged bleeding. HMB was particularly prevalent among patients with BP who were taking valproate (Vuong et al., 2014; Fu et al., 2020; Konicki et al., 2021). Serum testosterone increases induced by valproate may contribute to the development of HMB (Bilo and Meo, 2008; Flores-Ramos et al., 2020; Kenna et al., 2009; McAllister-Williams, 2006; O'Donovan et al., 2002; Rasgon et al., 2005b; Zhang et al., 2016). Notably, in 2018, Elboga et al. reported the case of a boy who had manic episode after being given testosterone replacement therapy for hypogonadotropic hypogonadism (Elboga and Sayiner, 2018).

The frequency of HMB periods experienced was found to be influenced by blood sugar levels, cumulative medication dosages, and HbA1c changes emerging within 2–3 months after accepting treatment. Indeed, HbA1c and age (<30 years) were found to be persistent risk factors for treatment-associated HMB across the diagnostic groups. Among patients with SCZ, hyper-prolactin, high TC, high triglyceride levels, and an overweight BMI before treatment were all found to be risk factors for subsequent HMB. It remains to be determined in prospective cohort studies whether blood sugar disturbances are causative of or consequential to HMB. Notwithstanding, these findings indicate that physicians managing the cases of young women with mental illnesses should be attentive to changes in HbA1c and blood sugar, particularly in relation to monitoring cumulative medication dosage and medication phases.

Although patients with menstrual dysfunction of any kind in our study were found to have elevated levels of prolactin, estrogen, progesterone, and testosterone, those with HMB per se did not have significantly elevated levels, and the frequency of HMB periods did not correlate with prolactin levels, consistent with previous studies (Lethaby et al., 2015). The need for a gynecological intervention was also not found to be related to prolactin level, neither was it related to estrogen, progesterone, or testosterone levels.

Medication-induced menstrual dysfunction could be due to drug-induced disruption of the hypothalamic-pituitary-ovarian (H-P-O) axis, thus altering the estrogen and progesterone cycles that regulate menstruation (Kadir and Davies, 2013; Bradley and Gueye, 2016; James, 2016; Ryan, 2017; Thomas et al., 2020; Ramalho et al., 2021). HMB may follow several months of amenorrhea/oligomenorrhea during which the endometrium could not fall off normally, which can cause endometrial hyperplasia (Kadir and Davies, 2013; Bradley and Gueye, 2016; James, 2016; Thomas et al., 2020; Ramalho et al., 2021). According to this view, HMB may reflect a disorder of estrogen and progesterone secretion, independent of hyper-prolactin. In women with BP, valproate has been reported to induce hyperandrogenism, which leads to oligomenorrhea, consistent with an H-P-O disturbance (Death et al., 2005; Joffe et al., 2006a; Bilo and Meo, 2008; Sidhu et al., 2018). Notwithstanding, pharmacotherapeutic-induced hyper-prolactin reflects a cryptorrhea phenomenon, the effects of which should be elucidated in a prospective cohort study. Psychiatric medications have secondary effects on the hemic system (Dahl, 1986; Krieger et al., 2004; Dietrich-Muszalska and Wachowicz, 2017; Pavlidi et al., 2021), and thus can cause or exacerbate coagulation disorders and abnormal bleeding, which can lead to HMB in women (Yasui-Furukori et al., 2012; Kranz et al., 2021). Although the precise mechanisms underlying these drug effects are unknown, physicians should be screening for HMB in the course of female psychiatric patient monitoring.

Surprisingly, among the cognitive functions followed, impaired visual learning performance emerged as being strongly associated with HMB. The reasons for this association are difficult to speculate about, but certainly worthy of future examination.

Lithium was unique among the analyzed medications in that it seemed to be a protective factor against HMB. Interestingly, lithium has also been shown to be a protective factor against cognitive impairment (Matsunaga et al., 2015; Ochoa, 2022). However, to the best of our knowledge, lithium effects on cognitive performance cannot explain its protective influence on HMB in women with severe mental illnesses.

HMB awareness among psychiatrists was found to be abysmal at 2.28%, particularly given the substantial prevalence of HMB in the patient population served by the surveyed psychiatrists. Common remedies for menstrual dysfunction, including aripiprazole and traditional Chinese medicines, were ineffective for alleviating HMB in our patient sample. Thus, there is an urgent need to alert psychiatrists of this epidemiological information, especially those who treat women with BP.

This study had a number of limitations that warrant discussion. First, it was a retrospective study employing the PABC to assess HMB history. The validity of the PBAC for assessing menstrual bleeding in prior months needs to be confirmed. The patients in this sample were confirmed to have a good memory according to the Wechsler memory scale and used the last menstrual bleeding status as a reference standard.

Second, although our data pointed to blood sugar variables, including HbA1c, as risk factors for HMB. Even HbA1c, which can only reflect blood sugar alterations over the preceding 3 months, cannot reflect 18 months of physiological history. Although our data support the view that HbA1c alterations may trigger HMB (Sharawy et al., 2016; van Baar et al., 2022), the mechanisms of such an effect, if true, remain to be clarified.

Third, although valproate use, theoretically, might explain the observed higher incidence of HMD in BP patients than in the MDD and SCZ groups due to valproate disruption of the H-P-O axis, nearly half of the patients in the MDD and SCZ groups were using valproate as a synergist. In a prior study of patients with SCZ, antipsychotic agents were not significantly related to testosterone or estradiol levels (O'Donovan et al., 2002). Although antidepressants cannot induce testosterone upregulation, testosterone has been reported to have an antidepressive effect by way of its reducing influence on monoamine oxidase A levels ^88^. Related to this concern, as discussed above, our data do not enable us to disentangle how high blood sugar levels and hyperprolactinemia may influence HMB risk via effects on the H-P-O-axis. Thus, there are as yet to be clarified sophisticated relationships among therapeutic agents and H-P-O axis pathways.

Although obesity has been previously associated with HMB risk (Seif et al., 2015), the present data cannot confirm this putative relationship because antipsychotic medication use itself was associated with increasing BMI in women with SCZ. Additionally, we did not assess HMB prior to mental illness onset. Although we did not find an association between ECT and HMB, a minor portion of our sample received ECT and thus we are not confident in ruling out a possible association.

Notably, the remedies recommended to our patients for menstrual dysfunction, primarily aripiprazole and traditional Chinese medicines did not normalize menstrual function. Indeed, remarkably, every individual in our sample (*N* = 3,094) reported having irregular menstrual periods. Finally, the results obtained in the present treatment-resistant patient sample may not generalize to treatment response patients; only ∼30% of patients with BP, MDD, or SCZ (beyond this study) are treatment resistant.

## Conclusion

The present study yielded five pivotal pieces of clinical reference information. 1) The risk for HMB in young adult women is substantial. 2) Psychiatric medications may induce hyperglycemia and poor visual learning performance within three treatment months, and medication dosage is related to HMB risk. Thus, healthcare providers should be screening for the emergence of HMB and adjust treatment plans accordingly. 3) Women with BP who are treated with valproate are at heightened risk of HMB, suggesting that perhaps valproate therapy should be prescribed less frequently for BP, at least in young women. 4) Lithium is a protective factor against HMB. Finally, 5) psychiatrists’ awareness of HMB risk in women with severe mental illness is extremely low. Hence, there is a need to inform psychiatric clinicians of the need to pay attention to HMB risk.

## Data Availability

The original contributions presented in the study are included in the article/supplementary material, further inquiries can be directed to the corresponding authors.

## References

[B1] An H. Du X. Huang X. Qi L. Jia Q. Yin Q. (2018). Obesity, Altered Oxidative Stress, and Clinical Correlates in Chronic Schizophrenia Patients. Transl. Psychiatry 8, 258. 10.1038/s41398-018-0303-7 30498208 PMC6265271

[B2] APA (2016). Psychiatric Services in Correctional Facilities. Third Edition. Virginia, US: American Psychiatric Association. 978-970-89042-89464-89043.

[B3] APA, American Psychiatric Association (1994). Diagnostic and Statistical Manual of Mental Disorders. 4th ed. Virginia, US: American Psychiatric Association.

[B4] Aranth J. Lindberg C. (1992). Bleeding, a Side Effect of Fluoxetine. Am. J. Psychiatry 149, 412. 10.1176/ajp.149.3.412a 1536285

[B5] Arias-de la Torre J. Ronaldson A. Prina M. Matcham F. Pinto Pereira S. M. Hatch S. L. (2021). Depressive Symptoms During Early Adulthood and the Development of Physical Multimorbidity in the UK: An Observational Cohort Study. Lancet Healthy Longev. 2, e801–e810. 10.1016/S2666-7568(21)00259-2 34901908 PMC8636278

[B6] Baandrup L. (2020). Polypharmacy in Schizophrenia. Basic Clin. Pharmacol. Toxicol. 126, 183–192. 10.1111/bcpt.13384 31908124

[B7] Barnett R. (2018). Schizophrenia. Lancet 391, 648. 10.1016/S0140-6736(18)30237-X 29617256

[B8] Beck A. T. Baruch E. Balter J. M. Steer R. A. Warman D. M. (2004). A New Instrument for Measuring Insight: The Beck Cognitive Insight Scale. Schizophr. Res. 68, 319–329. 10.1016/S0920-9964(03)00189-0 15099613

[B9] Bekhbat M. Chu K. Le N. A. Woolwine B. J. Haroon E. Miller A. H. (2018). Glucose and Lipid-Related Biomarkers and the Antidepressant Response to Infliximab in Patients with Treatment-Resistant Depression. Psychoneuroendocrinology 98, 222–229. 10.1016/j.psyneuen.2018.09.004 30249443 PMC6214671

[B10] Besag F. M. C. Vasey M. J. Salim I. (2021). Is Adjunct Aripiprazole Effective in Treating Hyperprolactinemia Induced by Psychotropic Medication? A Narrative Review. CNS Drugs 35, 507–526. 10.1007/s40263-021-00812-1 33880739

[B11] Bilo L. Meo R. (2008). Polycystic Ovary Syndrome in Women Using Valproate: a Review. Gynecol. Endocrinol. 24, 562–570. 10.1080/09513590802288259 19012099

[B12] Birchwood M. Smith J. Drury V. Healy J. Macmillan F. Slade M. (1994). A Self-Report Insight Scale for Psychosis: Reliability, Validity and Sensitivity to Change. Acta Psychiatr. Scand. 89, 62–67. 10.1111/j.1600-0447.1994.tb01487.x 7908156

[B13] Bo Q. Xing X. Li T. Mao Z. Zhou F. Wang C. (2021). Menstrual Dysfunction in Women with Schizophrenia During Risperidone Maintenance Treatment. J. Clin. Psychopharmacol. 41, 135–139. 10.1097/JCP.0000000000001344 33538534

[B14] Bora E. Akdede B. B. Alptekin K. (2017). The Relationship Between Cognitive Impairment in Schizophrenia and Metabolic Syndrome: A Systematic Review and Meta-Analysis. Psychol. Med. 47, 1030–1040. 10.1017/S0033291716003366 28032535

[B15] Borbély É. Simon M. Fuchs E. Wiborg O. Czéh B. Helyes Z. (2022). Drug Developmental Strategies for Treatment-Resistant Depression. Br. J. Pharmacol. 179, 1146–1186. 10.1111/bph.15753 34822719 PMC9303797

[B16] Bowie R. C. Best M. W. Depp C. Mausbach T. B. Patterson T. L. Pulver A. E. (2018). Cognitive and Functional Deficits in Bipolar Disorder and Schizophrenia as a Function of the Presence and History of Psychosis. Bipolar Disord. 20 (7), 604–613. 10.1111/bdi.12654 29777563

[B17] Bradley L. D. Gueye N. A. (2016). The Medical Management of Abnormal Uterine Bleeding in Reproductive-Aged Women. Am. J. Obstet. Gynecol. 214, 31–44. 10.1016/j.ajog.2015.07.044 26254516

[B18] Calaf J. Cancelo M. J. Andeyro M. Jiménez J. M. Perelló J. Correa M. (2020). Development and Psychometric Validation of a Screening Questionnaire to Detect Excessive Menstrual Blood Loss that Interferes in Quality of Life: The SAMANTA Questionnaire. J. Womens Health (Larchmt) 29, 1021–1031. 10.1089/jwh.2018.7446 32580622

[B19] Chen H. Wang X. T. Bo Q. G. Zhang D. M. Qi Z. B. Liu X. (2017). Menarche, Menstrual Problems and Suicidal Behavior in Chinese Adolescents. J. Affect Disord. 209, 53–58. 10.1016/j.jad.2016.11.027 27886570

[B20] Culpepper L. Lam R. W. McIntyre R. S. (2017). Cognitive Impairment in Patients with Depression: Awareness, Assessment, and Management. J. Clin. Psychiatry 78, 1383–1394. 10.4088/JCP.tk16043ah5c 29345866

[B21] Dahl S. G. (1986). Plasma Level Monitoring of Antipsychotic Drugs. Clinical Utility. Clin. Pharmacokinet. 11, 36–61. 10.2165/00003088-198611010-00003 2868820

[B22] Davies J. Kadir R. A. (2017). Heavy Menstrual Bleeding: An Update on Management. Thromb. Res. 151 (Suppl. 1), S70–s77. 10.1016/S0049-3848(17)30072-5 28262240

[B23] Death A. K. McGrath K. C. Handelsman D. J. (2005). Valproate Is an Anti-Androgen and Anti-Progestin. Steroids 70, 946–953. 10.1016/j.steroids.2005.07.003 16165177

[B24] Dietrich-Muszalska A. Wachowicz B. (2017). Platelet Haemostatic Function in Psychiatric Disorders: Effects of Antidepressants and Antipsychotic Drugs. World J. Biol. Psychiatry 18, 564–574. 10.3109/15622975.2016.1155748 27112326

[B25] El-Nashar S. A. Hopkins M. R. Barnes S. A. Pruthi R. K. Gebhart J. B. Cliby W. A. (2010). Health-Related Quality of Life and Patient Satisfaction after Global Endometrial Ablation for Menorrhagia in Women with Bleeding Disorders: A Follow-Up Survey and Systematic Review. Am. J. Obstet. Gynecol. 202, 348. 10.1016/j.ajog.2009.11.032 20060089

[B26] Elboga G. Sayiner Z. A. (2018). Rare Cause of Manic Period Trigger in Bipolar Mood Disorder: Testosterone Replacement. BMJ Case Rep. 2018, bcr2018225108. 10.1136/bcr-2018-225108 PMC6078280 30076163

[B27] Elias A. Thomas N. Sackeim H. A. (2021). Electroconvulsive Therapy in Mania: A Review of 80 Years of Clinical Experience. Am. J. Psychiatry 178, 229–239. 10.1176/appi.ajp.2020.20030238 33167675

[B28] First M. B. Spitzer R. L. Gibbon M. Williams J. B. W. (1996). Structured Clinical Interview for DSM-IV Axis I Disorders, Clinician Version (SCID-CV). Washington, DC: American Psychiatric Press, Inc.

[B29] Flores-Ramos M. Becerra-Palars C. Hernández González C. Chavira R. Bernal-Santamaría N. Martínez Mota L. (2020). Serum Testosterone Levels in Bipolar and Unipolar Depressed Female Patients and the Role of Medication Status. Int. J. Psychiatry Clin. Pract. 24, 53–58. 10.1080/13651501.2019.1680696 32096661

[B30] Fu L. H. Schwartz J. Moy A. Knaplund C. Kang M. J. Schnock K. O. (2020). Development and Validation of Early Warning Score System: A Systematic Literature Review. J. Biomed. Inf. 105, 103410. 10.1016/j.jbi.2020.103410 PMC7295317 32278089

[B31] García-Carmona J. A. Simal-Aguado J. Campos-Navarro M. P. Valdivia-Muñoz F. Galindo-Tovar A. (2021). Evaluation of Long-Acting Injectable Antipsychotics with the Corresponding Oral Formulation in a Cohort of Patients with Schizophrenia: A Real-World Study in Spain. Int. Clin. Psychopharmacol. 36, 18–24. 10.1097/yic.0000000000000339 33086252

[B32] Gu L. Huang J. Tan J. Wei Q. Jiang H. Shen T. (2016). Impact of TLR5 Rs5744174 on Stroke Risk, Gene Expression and on Inflammatory Cytokines, and Lipid Levels in Stroke Patients. Neurol. Sci. 37, 1537–1544. 10.1007/s10072-016-2607-9 27262705

[B33] Haddad P. M. Wieck A. (2004). Antipsychotic-Induced Hyperprolactinaemia: Mechanisms, Clinical Features and Management. Drugs 64, 2291–2314. 10.2165/00003495-200464200-00003 15456328

[B34] Hayasaka Y. Purgato M. Magni L. R. Ogawa Y. Takeshima N. Cipriani A. (2015). Dose Equivalents of Antidepressants: Evidence-Based Recommendations from Randomized Controlled Trials. J. Affect Disord. 180, 179–184. 10.1016/j.jad.2015.03.021 25911132

[B35] Hidalgo B. Goodman M. (2013). Multivariate or Multivariable Regression? Am. J. Public Health 103, 39–40. 10.2105/AJPH.2012.300897 23153131 PMC3518362

[B36] Howes O. D. Thase M. E. Pillinger T. (2021). Treatment Resistance in Psychiatry: State of the Art and New Directions. Mol. Psychiatry 27, 58–72. 10.1038/s41380-021-01200-3 34257409 PMC8960394

[B37] Huhn M. Nikolakopoulou A. Schneider-Thoma J. Krause M. Samara M. Peter N. (2019). Comparative Efficacy and Tolerability of 32 Oral Antipsychotics for the Acute Treatment of Adults with Multi-Episode Schizophrenia: A Systematic Review and Network Meta-Analysis. Lancet 394, 939–951. 10.1016/S0140-6736(19)31135-3 31303314 PMC6891890

[B38] Hui Poon S. Sim K. Baldessarini R. J. (2015). Pharmacological Approaches for Treatment-Resistant Bipolar Disorder. Curr. Neuropharmacol. 13, 592–604. 10.2174/1570159x13666150630171954 26467409 PMC4761631

[B39] Iles S. Gath D. (1989). Psychological Problems and Uterine Bleeding. Baillieres Clin. Obstet. Gynaecol. 3, 375–389. 10.1016/s0950-3552(89)80028-8 2692926

[B40] James A. H. (2016). Heavy Menstrual Bleeding: Work-Up and Management. Hematol. Am. Soc. Hematol. Educ. Program 2016, 236–242. 10.1182/asheducation-2016.1.236 PMC6142441 27913486

[B41] Jang E. H. Lee J. H. Kim S. A. (2021). Acute Valproate Exposure Induces Mitochondrial Biogenesis and Autophagy with FOXO3a Modulation in SH-Sy5y Cells. Cells 10, 2522. 10.3390/cells10102522 34685502 PMC8533738

[B42] Joffe H. Cohen L. S. Suppes T. McLaughlin W. L. Lavori P. Adams J. M. (2006a). Valproate Is Associated with New-Onset Oligoamenorrhea with Hyperandrogenism in Women with Bipolar Disorder. Biol. Psychiatry 59, 1078–1086. 10.1016/j.biopsych.2005.10.017 16448626

[B43] Joffe H. Kim D. R. Foris J. M. Baldassano C. F. Gyulai L. Hwang C. H. (2006b). Menstrual Dysfunction Prior to Onset of Psychiatric Illness Is Reported More Commonly by Women with Bipolar Disorder Than by Women with Unipolar Depression and Healthy Controls. J. Clin. Psychiatry 67, 297–304. 10.4088/jcp.v67n0218 16566627

[B44] Kadir R. A. Davies J. (2013). Hemostatic Disorders in Women. J. Thromb. Haemost. 11 (Suppl. 1), 170–179. 10.1111/jth.12267 23809121

[B45] Kenna H. A. Jiang B. Rasgon N. L. (2009). Reproductive and Metabolic Abnormalities Associated with Bipolar Disorder and its Treatment. Harv Rev. Psychiatry 17, 138–146. 10.1080/10673220902899722 19373621

[B46] Kennedy J. L. Altar C. A. Taylor D. L. Degtiar I. Hornberger J. C. (2014). The Social and Economic Burden of Treatment-Resistant Schizophrenia: A Systematic Literature Review. Int. Clin. Psychopharmacol. 29, 63–76. 10.1097/YIC.0b013e32836508e6 23995856

[B47] Ko J. K. Y. Lao T. T. Cheung V. Y. T. (2021). Pictorial Blood Loss Assessment Chart for Evaluating Heavy Menstrual Bleeding in Asian Women. Hong Kong Med. J. 27, 399–404. 10.12809/hkmj208743 34949729

[B48] Konicki W. Soletic L. C. Karlis V. Aaron C. (2021). Point-of-Care Pregnancy Testing in Outpatient Sedation Anesthesia: Experience from an Urban Hospital-Based Oral and Maxillofacial Surgery Clinic. J. Oral Maxillofac. Surg. 79, 2444–2447. 10.1016/j.joms.2021.05.013 34153249

[B49] Kranz G. S. Spies M. Vraka C. Kaufmann U. Klebermass E. M. Handschuh P. A. (2021). High-Dose Testosterone Treatment Reduces Monoamine Oxidase A Levels in the Human Brain: A Preliminary Report. Psychoneuroendocrinology 133, 105381. 10.1016/j.psyneuen.2021.105381 34416504

[B50] Krieger K. Klimke A. Henning U. (2004). Antipsychotic Drugs Influence Transport of the Beta-Adrenergic Antagonist [3H]-Dihydroalprenolol into Neuronal and Blood Cells. World J. Biol. Psychiatry 5, 100–106. 10.1080/15622970410029918 15179669

[B51] Kumar A. Datta S. S. Wright S. D. Furtado V. A. Russell P. S. (2013). Atypical Antipsychotics for Psychosis in Adolescents. Cochrane Database Syst. Rev. 15, Cd009582. 10.1002/14651858.cd009582.pub2 PMC11339588 24129841

[B52] Lee J. Kim M. Chae H. Kim Y. Park H. I. Kim Y. (2013). Evaluation of Enzymatic BM Test HbA1c on the JCA-Bm6010/C and Comparison with Bio-Rad Variant II Turbo, Tosoh HLC 723 G8, and AutoLab Immunoturbidimetry Assay. Clin. Chem. Lab. Med. 51, 2201–2208. 10.1515/cclm-2013-0238 23898021

[B53] Lethaby A. Hickey M. Garry R. Penninx J. (2009). Endometrial Resection/Ablation Techniques for Heavy Menstrual Bleeding. Cochrane Database Syst. Rev. Cd001501 7, CD001501. 10.1002/14651858.cd001501.pub3 19821278

[B54] Lethaby A. Hussain M. Rishworth J. R. Rees M. C. (2015). Progesterone or Progestogen-Releasing Intrauterine Systems for Heavy Menstrual Bleeding. Cochrane Database Syst. Rev. 30, Cd002126. 10.1002/14651858.cd002126.pub3 25924648

[B55] Leucht S. Wahlbeck K. Hamann J. Kissling W. (2003). New Generation Antipsychotics versus Low-Potency Conventional Antipsychotics: A Systematic Review and Meta-Analysis. Lancet 361, 1581–1589. 10.1016/S0140-6736(03)13306-5 12747876

[B57] Lo B. S. Szuhany K. L. Kredlow M. A. Wolfe R. Mueser K. T. McGurk S. R. (2016). A Confirmatory Factor Analysis of the MATRICS Consensus Cognitive Battery in Severe Mental Illness. Schizophr. Res. 175 (1–3), 79–84. 10.1016/j.schres.2016.03.013 27041675

[B58] Matsunaga S. Kishi T. Annas P. Basun H. Hampel H. Iwata N. (2015). Lithium as a Treatment for Alzheimer's Disease: A Systematic Review and Meta-Analysis. J. Alzheimers Dis. 48, 403–410. 10.3233/JAD-150437 26402004

[B59] McAllister-Williams R. H. (2006). Relapse Prevention in Bipolar Disorder: A Critical Review of Current Guidelines. J. Psychopharmacol. 20, 12–16. 10.1177/1359786806063071 16551667

[B60] McCleery A. Green M. F. Hellemann. G. S. Baade L. E. Gold G. M. Keefe R. S. E. (2015). Latent Structure of Cognition in Schizophrenia: A Confirmatory Factor Analysis of the MATRICS Consensus Cognitive Battery (MCCB) Psychol. Med 45 (12), 2657–2666. 10.1017/S0033291715000641 PMC4523424 25916421

[B62] McIntyre R. S. Berk M. Brietzke E. Goldstein B. I. López-Jaramillo C. Kessing L. V. (2020). Bipolar Disorders. Lancet 396, 1841–1856. 10.1016/S0140-6736(20)31544-0 33278937

[B63] Meyer J. M. (2002). A Retrospective Comparison of Weight, Lipid, and Glucose Changes Between Risperidone- and Olanzapine-Treated Inpatients: Metabolic Outcomes After 1 Year. J. Clin. Psychiatry 63, 425–433. 10.4088/jcp.v63n0509 12019668

[B64] Mizuno Y. Suzuki T. Nakagawa A. Yoshida K. Mimura M. Fleischhacker W. W. (2014). Pharmacological Strategies to Counteract Antipsychotic-Induced Weight Gain and Metabolic Adverse Effects in Schizophrenia: A Systematic Review and Meta-Analysis. Schizophr. Bull. 40, 1385–1403. 10.1093/schbul/sbu030 24636967 PMC4193713

[B65] Muir C. C. Treasurywala K. McAllister S. Sutherland J. Dukas L. Berger R. G. (2008). Enzyme Immunoassay of Testosterone, 17beta-Estradiol, and Progesterone in Perspiration and Urine of Preadolescents and Young Adults: Exceptional Levels in Men's Axillary Perspiration. Horm. Metab. Res. 40, 819–826. 10.1055/s-0028-1082042 18711693

[B66] Nick T. G. Campbell K. M. (2007). Logistic Regression. Methods Mol. Biol. 404, 273–301. 10.1007/978-1-59745-530-5_14 18450055

[B67] Nikolac Gabaj N. Miler M. Vrtarić A. Hemar M. Filipi P. Kocijančić M. (2018). Precision, Accuracy, Cross Reactivity and Comparability of Serum Indices Measurement on Abbott Architect C8000, Beckman Coulter AU5800 and Roche Cobas 6000 C501 Clinical Chemistry Analyzers. Clin. Chem. Lab. Med. 56, 776–788. 10.1515/cclm-2017-0889 29315074

[B68] Nikoloulopoulos A. K. (2012). A Multivariate Logistic Regression. Biostatistics 13, 1–3. 10.1093/biostatistics/kxr014 21669860

[B69] Noerpramana N. P. (1997). Blood-Lipid Fractions: the Side-Effects and Continuation of Norplant Use. Adv. Contracept. 13, 13–37. 10.1023/a:1006512211238 9181182

[B70] O'Donovan C. Kusumakar V. Graves G. R. Bird D. C. (2002). Menstrual Abnormalities and Polycystic Ovary Syndrome in Women Taking Valproate for Bipolar Mood Disorder. J. Clin. Psychiatry 63, 322–330. 10.4088/jcp.v63n0409 12000206

[B71] Ochoa E. L. M. (2022). Lithium as a Neuroprotective Agent for Bipolar Disorder: An Overview. Cell. Mol. Neurobiol. 42, 85–97. 10.1007/s10571-021-01129-9 34357564 PMC11441275

[B72] Paavola J. T. Väntti N. Junkkari A. Huttunen T. J. von Und Zu Fraunberg M. Koivisto T. (2019). von Und Zu Fraunberg Antipsychotic Use Among 1144 Patients After Aneurysmal Subarachnoid Hemorrhage. Stroke 50, 1711–1718. 10.1161/strokeaha.119.024914 31167617

[B73] Padda J. Khalid K. Hitawala G. Batra N. Pokhriyal S. Mohan A. (2021). Depression and its Effect on the Menstrual Cycle. Cureus 13, e16532. 10.7759/cureus.16532 34430141 PMC8378322

[B74] Papakostas G. I. Miller K. K. Petersen T. Sklarsky K. G. Hilliker S. E. Klibanski A. (2006). Serum Prolactin Levels Among Outpatients with Major Depressive Disorder During the Acute Phase of Treatment with Fluoxetine. J. Clin. Psychiatry 67, 952–957. 10.4088/jcp.v67n0612 16848655

[B75] Papp M. Cubala W. J. Swiecicki L. Newman-Tancredi A. Willner P. (2021). Perspectives for Therapy of Treatment-Resistant Depression. Br. J. Pharmacol. 10.1111/bph.15596 34128229

[B76] Paulzen M. Haen E. Stegmann B. Hiemke C. Gründer G. Lammertz S. E. (2016). Body Mass Index (BMI) but Not Body Weight Is Associated with Changes in the Metabolism of Risperidone; A Pharmacokinetics-Based Hypothesis. Psychoneuroendocrinology 73, 9–15. 10.1016/j.psyneuen.2016.07.009 27448523

[B77] Pavlidi P. Kokras N. Dalla C. (2021). Antidepressants' Effects on Testosterone and Estrogens: What Do We Know? Eur. J. Pharmacol. 899, 173998. 10.1016/j.ejphar.2021.173998 33676942

[B78] Piatoikina A. S. Lyakhova A. A. Semennov I. V. Zhilyaeva T. V. Kostina O. V. Zhukova E. S. (2022). Association of Antioxidant Deficiency and the Level of Products of Protein and Lipid Peroxidation in Patients with the First Episode of Schizophrenia. J. Mol. Neurosci. 72 (2), 217–225. 10.1007/s12031-021-01884-w 34410570

[B79] Pottegård A. Lash T. L. Cronin-Fenton D. Ahern T. P. Damkier P. (2018). Use of Antipsychotics and Risk of Breast Cancer: A Danish Nationwide Case-Control Study. Br. J. Clin. Pharmacol. 84, 2152–2161. 10.1111/bcp.13661 29858518 PMC6089812

[B80] Pramodh S. (2020). Exploration of Lifestyle Choices, Reproductive Health Knowledge, and Polycystic Ovary Syndrome (PCOS) Awareness Among Female Emirati University Students. Int. J. Womens Health 12, 927–938. 10.2147/IJWH.S272867 33149703 PMC7604941

[B81] Ramalho I. Leite H. Águas F. (2021). Abnormal Uterine Bleeding in Adolescents: A Multidisciplinary Approach. Acta Med. Port. 34, 291–297. 10.20344/amp.12829 34214420

[B82] Rasgon N. L. Altshuler L. L. Fairbanks L. Elman S. Bitran J. Labarca R. (2005a). Reproductive Function and Risk for PCOS in Women Treated for Bipolar Disorder. Bipolar Disord. 7, 246–259. 10.1111/j.1399-5618.2005.00201.x 15898962

[B83] Rasgon N. L. Reynolds M. F. Elman S. Saad M. Frye M. A. Bauer M. (2005b). Longitudinal Evaluation of Reproductive Function in Women Treated for Bipolar Disorder. J. Affect Disord. 89, 217–225. 10.1016/j.jad.2005.08.002 16171873

[B84] Reavey J. J. Walker C. Murray A. A. Brito-Mutunayagam S. Sweeney S. Nicol M. (2021a). Obesity Is Associated with Heavy Menstruation that May Be Due to Delayed Endometrial Repair. J. Endocrinol. 249, 71–82. 10.1530/JOE-20-0446 33836495 PMC8052524

[B85] Reavey J. J. Walker C. Nicol M. Murray A. A. Critchley H. O. D. Kershaw L. E. (2021b). Markers of Human Endometrial Hypoxia Can Be Detected *In Vivo* and *Ex Vivo* During Physiological Menstruation. Hum. Reprod. 36, 941–950. 10.1093/humrep/deaa379 33496337 PMC7970728

[B86] Reynolds M. F. Sisk E. C. Rasgon N. L. (2007). Valproate and Neuroendocrine Changes in Relation to Women Treated for Epilepsy and Bipolar Disorder: A Review. Curr. Med. Chem. 14, 2799–2812. 10.2174/092986707782360088 18045126

[B88] Rossetti A. O. Alvarez V. (2021). Update on the Management of Status Epilepticus. Curr. Opin. Neurol. 34, 172–181. 10.1097/WCO.0000000000000899 33664203

[B89] Ryan S. A. (2017). The Treatment of Dysmenorrhea. Pediatr. Clin. North Am. 64, 331–342. 10.1016/j.pcl.2016.11.004 28292449

[B90] Rybak Y. E. Lai K. S. P. Ramasubbu R. Vila-Rodriguez F. Blumberger D. M. Chan P. (2021). Treatment-Resistant Major Depressive Disorder: Canadian Expert Consensus on Definition and Assessment. Depress Anxiety 38, 456–467. 10.1002/da.23135 33528865 PMC8049072

[B91] Schlaff W. D. Ackerman R. T. Al-Hendy A. Archer D. F. Barnhart K. T. Bradley L. D. (2020). Elagolix for Heavy Menstrual Bleeding in Women with Uterine Fibroids. N. Engl. J. Med. 382, 328–340. 10.1056/NEJMoa1904351 31971678

[B92] Schmidt M. (1996). Rey Auditory and Verbal Learning Test: A Handbook. California.Western Psychological Services.

[B93] Seif M. W. Diamond K. Nickkho-Amiry M. (2015). Obesity and Menstrual Disorders. Best. Pract. Res. Clin. Obstet. Gynaecol. 29, 516–527. 10.1016/j.bpobgyn.2014.10.010 25467426

[B94] Seppälä A. Pylvänäinen J. Lehtiniemi H. Hirvonen N. Corripio I. Koponen H. (2021). Predictors of Response to Pharmacological Treatments in Treatment-Resistant Schizophrenia - A Systematic Review and Meta-Analysis. Schizophr. Res. 236, 123–134. 10.1016/j.schres.2021.08.005 34496316

[B95] Shannon M. (1993). An Empathetic Look at Overweight. CCL Fam. Found. 20 (3), 5. 10.1016/0892-8967(93)90132-b 12318598

[B96] Shapley M. Croft P. R. McCarney R. Lewis M. (2000). Does Psychological Status Predict the Presentation in Primary Care of Women with a Menstrual Disturbance? Br. J. Gen. Pract. 50, 491–492. 10962793 PMC1313733

[B97] Sharawy M. H. El-Awady M. S. Megahed N. Gameil N. M. (2016). The Ergogenic Supplement β-Hydroxy-β-Methylbutyrate (HMB) Attenuates Insulin Resistance Through Suppressing GLUT-2 in Rat Liver. Can. J. Physiol. Pharmacol. 94, 488–497. 10.1139/cjpp-2015-0385 26871756

[B98] Sidhu H. S. Srinivasa R. Sadhotra A. (2018). Evaluate the Effects of Antiepileptic Drugs on Reproductive Endocrine System in Newly Diagnosed Female Epileptic Patients Receiving Either Valproate or Lamotrigine Monotherapy: A Prospective Study. Epilepsy Res. 139, 20–27. 10.1016/j.eplepsyres.2017.10.016 29144993

[B99] Sienaert P. Lambrichts L. Dols A. De Fruyt J. (2013). Evidence-based Treatment Strategies for Treatment-Resistant Bipolar Depression: A Systematic Review. Bipolar Disord. 15, 61–69. 10.1111/bdi.12026 23190379

[B100] Sinclair D. J. M. Zhao S. Qi F. Nyakyoma K. Kwong J. S. W. Adams C. E. (2019). Electroconvulsive Therapy for Treatment-Resistant Schizophrenia. Schizophr. Bull. 45, 730–732. 10.1093/schbul/sbz037 31150556 PMC6581135

[B102] Solé B. Jiménez E. Torrent C. Reinares M. Bonnin C. D. M. Torres I. (2017). Cognitive Impairment in Bipolar Disorder: Treatment and Prevention Strategies. Int. J. Neuropsychopharmacol. 20, 670–680. 10.1093/ijnp/pyx032 28498954 PMC5570032

[B103] Solmi M. Fornaro M. Ostinelli E. G. Zangani C. Croatto G. Monaco F. (2020). Safety of 80 Antidepressants, Antipsychotics, Anti-Attention-Deficit/Hyperactivity Medications and Mood Stabilizers in Children and Adolescents with Psychiatric Disorders: A Large Scale Systematic Meta-Review of 78 Adverse Effects. World Psychiatry 19, 214–232. 10.1002/wps.20765 32394557 PMC7215080

[B104] Stahl S. M. (2021). Stahl's Essential Psychopharmacology Neuroscientific Basis and Practical Applications. 5th edition. Cambridge, UK: Cambridge University Press.

[B105] Thomas W. Downes K. Desborough M. J. R. (2020). Bleeding of Unknown Cause and Unclassified Bleeding Disorders; Diagnosis, Pathophysiology and Management. Haemophilia 26, 946–957. 10.1111/hae.14174 33094877

[B106] Tian T. Wang D. Wei G. Wang J. Zhou H. Xu H. (2021). Prevalence of Obesity and Clinical and Metabolic Correlates in First-Episode Schizophrenia Relative to Healthy Controls. Psychopharmacol. 238 (3), 745–753. 10.1007/s00213-020-05727-1 33241480

[B107] Tondo L. Vázquez G. H. Baldessarini R. J. (2014). Options for Pharmacological Treatment of Refractory Bipolar Depression. Curr. Psychiatry Rep. 16, 431. 10.1007/s11920-013-0431-y 24425269

[B108] Trifu S. Sevcenco A. Stănescu M. Drăgoi A. M. Cristea M. B. (2021). Efficacy of Electroconvulsive Therapy as a Potential First-Choice Treatment in Treatment-Resistant Depression (Review). Exp. Ther. Med. 22, 1281. 10.3892/etm.2021.10716 34630636 PMC8461517

[B109] van Baar A. C. G. Devière J. Hopkins D. Crenier L. Holleman F. Galvão Neto M. P. (2022). Durable Metabolic Improvements 2 Years After Duodenal Mucosal Resurfacing (DMR) in Patients with Type 2 Diabetes (REVITA-1 Study). Diabetes Res. Clin. Pract. 184, 109194. 10.1016/j.diabres.2022.109194 35032562

[B110] Vuong K. McGeechan K. Armstrong B. K. Cust A. E. (2014). Risk Prediction Models for Incident Primary Cutaneous Melanoma: A Systematic Review. JAMA Dermatol 150, 434–444. 10.1001/jamadermatol.2013.8890 24522401

[B111] Walters J. Jones I. (2008). Clinical Questions and Uncertainty-Pprolactin Measurement in Patients with Schizophrenia and Bipolar Disorder. J. Psychopharmacol. 22, 82–89. 10.1177/0269881107086516 18477624

[B112] Wang J. Zou Y. Z. Cui J. F. Fan H. Z. Chen R. Chen N. (2015). Revision of the Wechsler Memory Scale-Fourth Edition of Chinese Version (Adult Battery). Chin. Ment. Health J. 29, 53–59. 10.3969/j.issn.1000-6729.2015.01.010

[B114] Yasui-Furukori N. Fujii A. Sugawara N. Tsuchimine S. Saito M. Hashimoto K. (2012). No Association Between Hormonal Abnormality and Sexual Dysfunction in Japanese Schizophrenia Patients Treated with Antipsychotics. Hum. Psychopharmacol. 27, 82–89. 10.1002/hup.1275 22249970

[B115] Zhang L. Li H. Li S. Zou X. (2016). Reproductive and Metabolic Abnormalities in Women Taking Valproate for Bipolar Disorder: A Meta-Analysis. Eur. J. Obstet. Gynecol. Reprod. Biol. 202, 26–31. 10.1016/j.ejogrb.2016.04.038 27160812

